# Evaluation of surface roughness of the bracket slot floor—a 3D perspective study

**DOI:** 10.1186/s40510-016-0116-2

**Published:** 2016-01-13

**Authors:** Chetankumar O. Agarwal, Ketan K. Vakil, Avinash Mahamuni, Pawankumar Dnyandeo Tekale, Prasad V. Gayake, Jeegar K. Vakil

**Affiliations:** Pune, Maharashtra India; Department of Orthodontics, S.M.B.T. Dental College and Hospital, Sangamner, Maharashtra India; Department of Orthodontics, Dr. Rajesh Ramdasji Kambe Dental College and Hospital, Akola, Maharashtra India

## Abstract

**Background:**

An important constituent of an orthodontic appliance is orthodontic brackets. It is either the bracket or the archwire that slides through the bracket slot, during sliding mechanics. Overcoming the friction between the two surfaces demands an important consideration in an appliance design. The present study investigated the surface roughness of four different commercially available stainless steel brackets.

**Methods:**

All tests were carried out to analyse quantitatively the morphological surface of the bracket slot floor with the help of scanning electron microscope (SEM) machine and to qualitatively analyse the average surface roughness (Sa) of the bracket slot floor with the help of a three-dimensional (3D) non-contact optical surface profilometer machine.

**Results:**

The SEM microphotographs were evaluated with the help of visual analogue scale, the surface roughness for group A = 0—very rough surface, group C = 1—rough surface, group B = 2—smooth surface, and group D = 3—very smooth surface. Surface roughness evaluation with the 3D non-contact optical surface profilometer machine was highest for group A, followed by group C, group B and group D. Groups B and D provided smooth surface roughness; however, group D had the very smooth surface with values 0.74 and 0.75 for mesial and distal slots, respectively.

**Conclusions:**

Evaluation of surface roughness of the bracket slot floor with both SEM and profilometer machine led to the conclusion that the average surface roughness was highest for group A, followed by group C, group B and group D.

## Background

One of the important constituents of an orthodontic appliance is brackets. Brackets stay for more time in the patient’s mouth, among all orthodontic materials [1]. In fixed orthodontic treatment, some degree of sliding between bracket and archwire occurs and the frictional resistance is encountered. So between the two surfaces, overcoming the friction demands an important consideration in an appliance design [2].

The frictional force to the force with which the contacting surfaces are pressed together is proportional and is affected by the nature of the surface at the interface (rough or smooth, chemically reactive or passive, modified by lubricants, etc.). This is because all surfaces, however smooth they are, have irregularities which are seen as large on a molecular scale, and real contact occurs at the peaks of the surface irregularities. These peaks, known as asperities, between the two surfaces carry the entire load [3, 4].

As compared to aesthetic ceramic brackets, metal brackets of stainless steel have a good superficial surface homogeneity and because of which, it has favourable mechanical properties and corrosion resistance. Even though a protective passive layer is present on the SS alloy, the Fe, Cr, or Ni (or all) ions may still be released from the metal surface in the acidic oral environment over the corrosion processes, which increases the risk of tissue damage, aesthetic changes (staining of the tooth by corrosive products) and loss of metal properties [5, 6].

Between bracket-wire interfaces, the friction occurs. Some force which is applied is dissipated as friction, and the remainder is being transferred to the supporting structures of the tooth to initiate tooth movement. So if the applied force is of sufficient magnitude, there is an occurrence of biological tooth response [4]. Hence, the amount of friction has a direct relation with the accuracy of bracket slot dimension and bracket slot roughness. This tempted us to evaluate the surface roughness of the bracket slot floor.

The ongoing appliance evolution resulted in two orthodontic bracket sizes that a clinician may choose either 0.018- or 0.022-in. slot. The 0.022-in.-slot size is larger as compared to 0.018-in. slot and facilitates easier wire insertion and less frictional binding during initial alignment and provides increased stiffness during retraction. McLaughlin, Bennett and Trevisi (MBT) itself recommends using 0.022-in. slot and also most of the orthodontists prefer using this slot size, so we decided to use 0.022-in. slot brackets for the study.

Hence, the present in vitro study was conducted to evaluate the surface roughness in three dimensions of the stainless steel bracket slot which can help to determine the clinical performance of the bracket, the accuracy of bracket slot dimension and roughness of the bracket slot.

### Aim and objectives

To access and compare the surface roughness of various as-received commercially available conventional 0.022 in. (0.56 mm) slot, MBT prescribed upper right 1st premolar stainless steel brackets.

## Methods

A total of 80 pre-adjusted conventional upper right first premolar stainless steel brackets in as-received condition with 0.022 in. (0.56 mm) slot and with MBT prescription (0° tip, −7° torque) from four different manufacturers were taken. In a sample size of 80 brackets, 20 brackets were allotted in each group as

- Group A: Gemini, 3M Unitek (Monrovia, CA)
- Group B: Mini 2000, Ormco Corp. (Glendora, CA)
- Group C: Opti-MIM, Ortho Organizers (San Marcos, CA)
- Group D: Mini master, American Orthodontics (Sheboygan, WI)

**Fig. 1:**
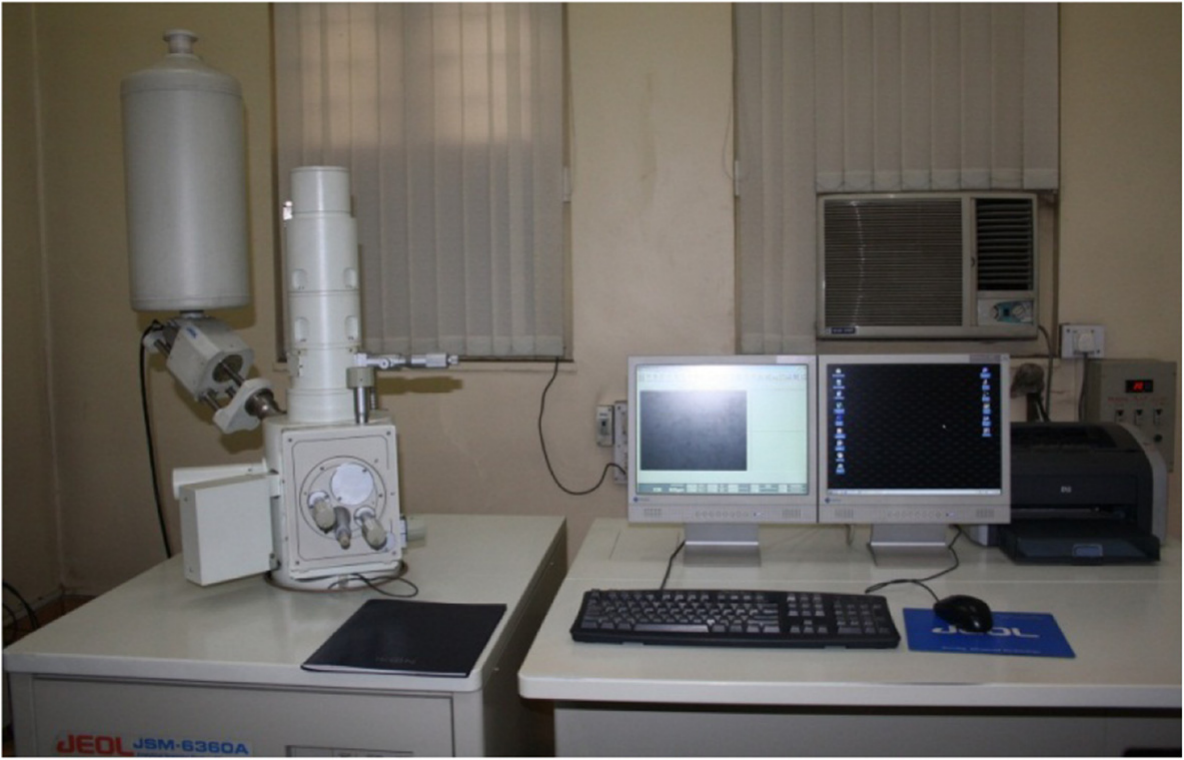
Scanning electron microscope (SEM) machine
- Three-dimensional (3D) non-contact optical surface profilometer machine (Taylor Hobson, England, available at Bangalore, India) (Fig. 2)

The following apparatus were used for the study:

- Scanning electron microscope (SEM) machine (JSM-6360A, JEOL, Japan, available at Pune, India, with 10–20 kV) (Fig. 1)

**Fig. 2:**
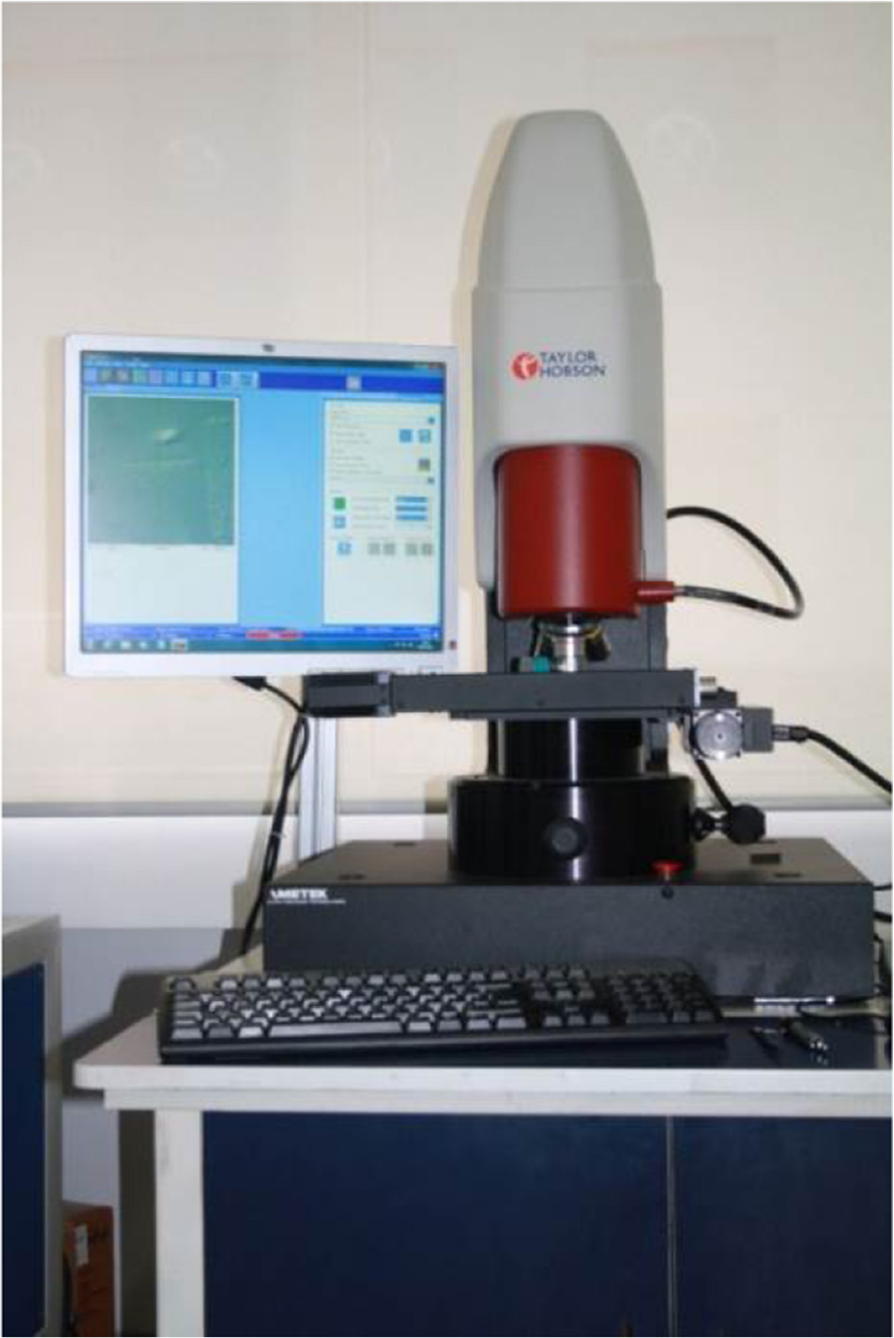
3D surface profilometer machine (Taylor Hobson)

### Procedure for scanning electron microscopy

**Fig. 3:**
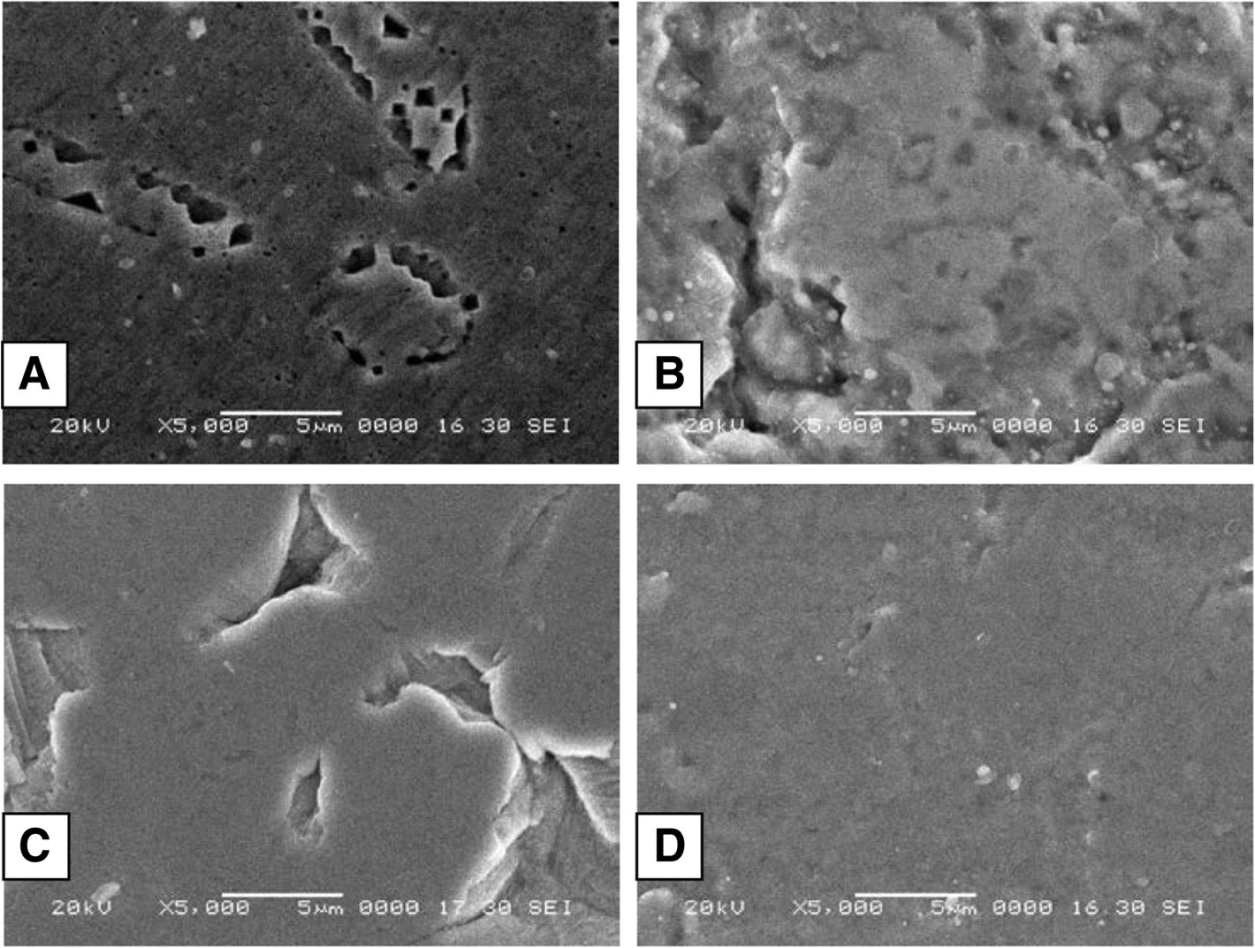
SEM microphotographs of stainless steel bracket slot from different manufacturers (×5000 magnification): **a** 3M Unitek, **b** Ormco, **c** Ortho Organizers, and **d** American Orthodontics

From the sample of as-received 80 brackets, 20 brackets—i.e. 5 brackets from each group (groups A, B, C, and D) were randomly selected to analyse the morphological surface of the bracket slot floor (mesial and distal slots), with the help of a SEM machine (Fig. 1). The surface was scanned and viewed on the monitor screen and representative microphotographs at ×5000 magnification [5] of each bracket slot floor were obtained, which was sufficient to have a clear view of the surface characteristics of the slot floor area (Fig. 3). The images which were obtained were saved for the visual evaluation, using a discrete scale quantitative classification containing four scores [6, 7]:

- 0—very rough surface
- 1—rough surface
- 2—smooth surface
- 3—very smooth surface

### Surface roughness measurement

Each group was evaluated for the Sa of the bracket slot floor with the help of a 3D non-contact optical surface profilometer machine (Fig. 2).

The measurements were done in sequence of the group’s name like group A was evaluated first then group B, C and D, respectively. Also, each time measurement of the mesial slot was done first and then the distal slot for all the samples to maintain a standardized protocol. Each sample was placed on the flat surface of the profilometer machine with the help of a tweezer, under the beam of the white light interferometry which is usually made up of the He-Ne, 633 nm [8].

### Configuration settings

#### Scanning speed

**Fig. 4:**
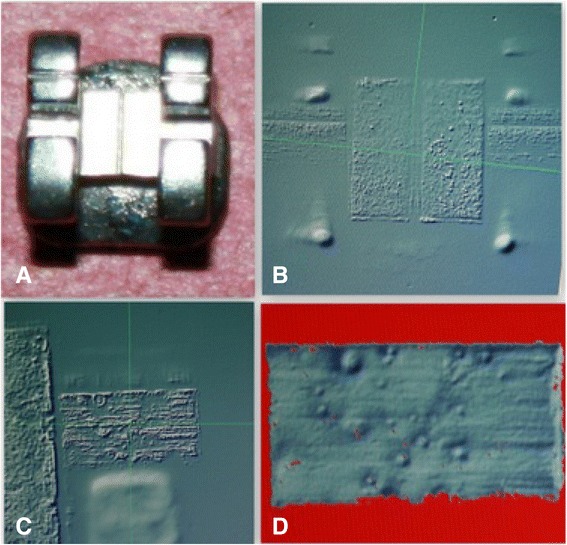
**a** Stainless steel bracket, **b** bracket view under the three-dimensional profiler machine, **c** distal slot view of the bracket, and **d** distal slot view of the bracket after scanning showing the scanned slot surface

The ×1 option was selected, such that the slower the scanning speed of the sample, the greater will be the details of the fringes of the bracket slot floor as shown in Fig. 4.

#### Measurement setting

**Fig. 5:**
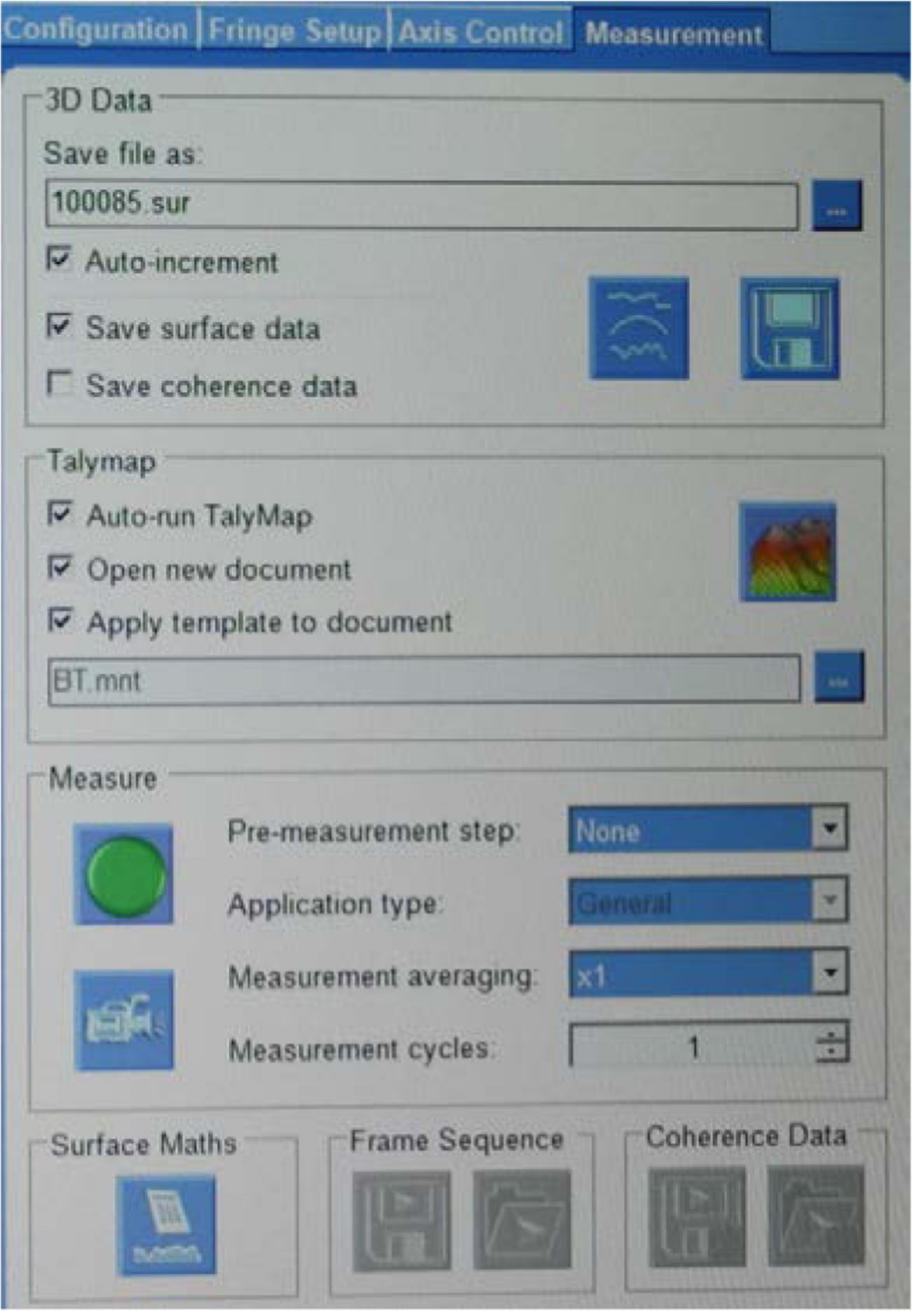
Measurement settings

**Fig. 6:**
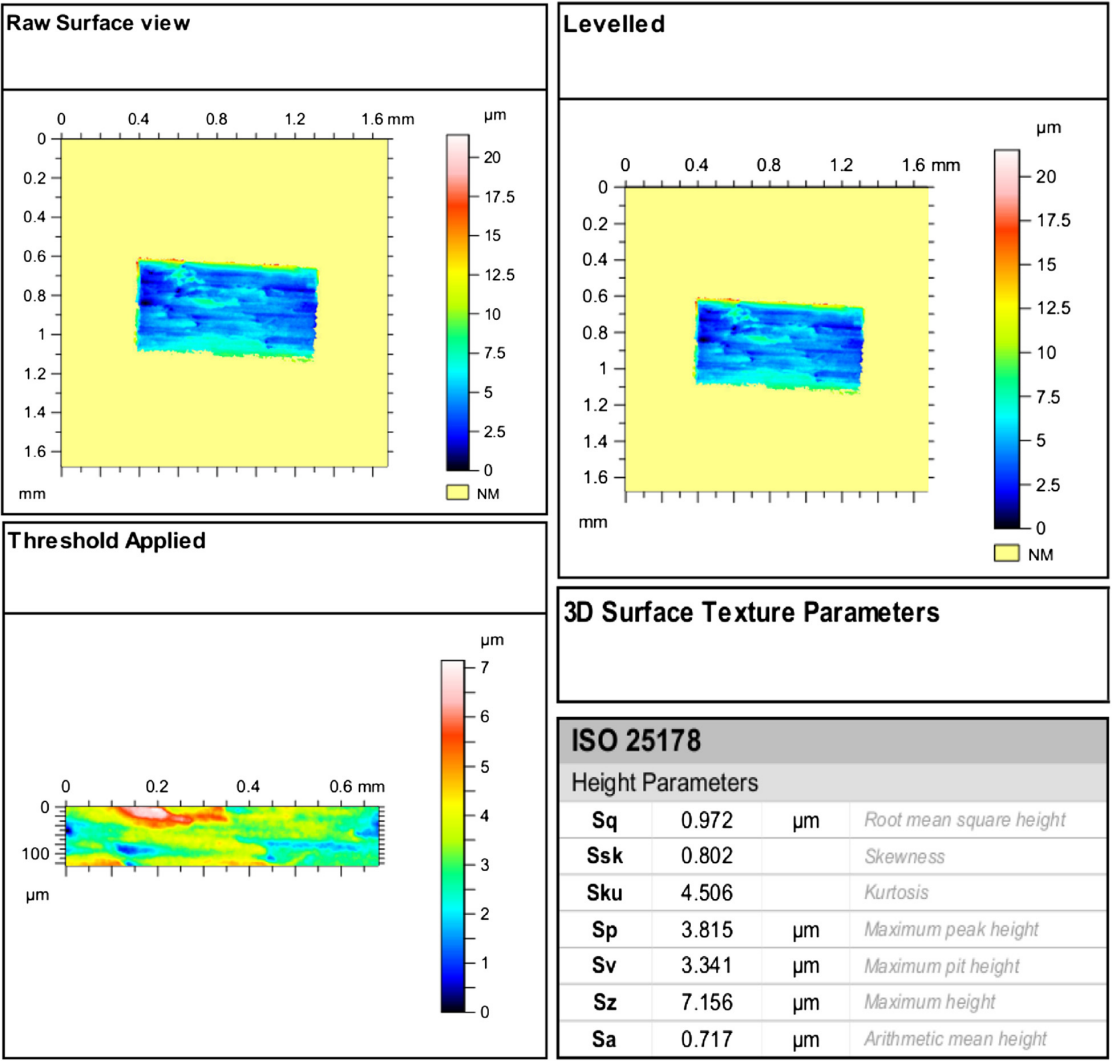
Steps in recording the 3D surface texture parameters. **a** Raw surface view, **b** levelled surface. **c** Threshold applied. **d**. Surface parameters

The measurement of the slot surface roughness is analysed by software Digital Surf, (TalyMap Platinum software, Leicester, England. Version no. 6.1.6001), from which we can receive 3D and 2D surface texture parameters (Fig. 5). The 3D surface texture parameters and height parameters (ISO25178) are the following (Fig. 6d):

- Sp—maximum peak height
- Sv—maximum pit height
- Sz—maximum height
- Sq—root mean square height
- Ssk—skewness
- Sku—kurtosis
- Sa—arithmetic mean height

From these 3D parameters, we are interested in the Sa value which is an average of the surface heights giving us the average surface roughness of the bracket slot floor in three dimensions.

**Fig. 7:**
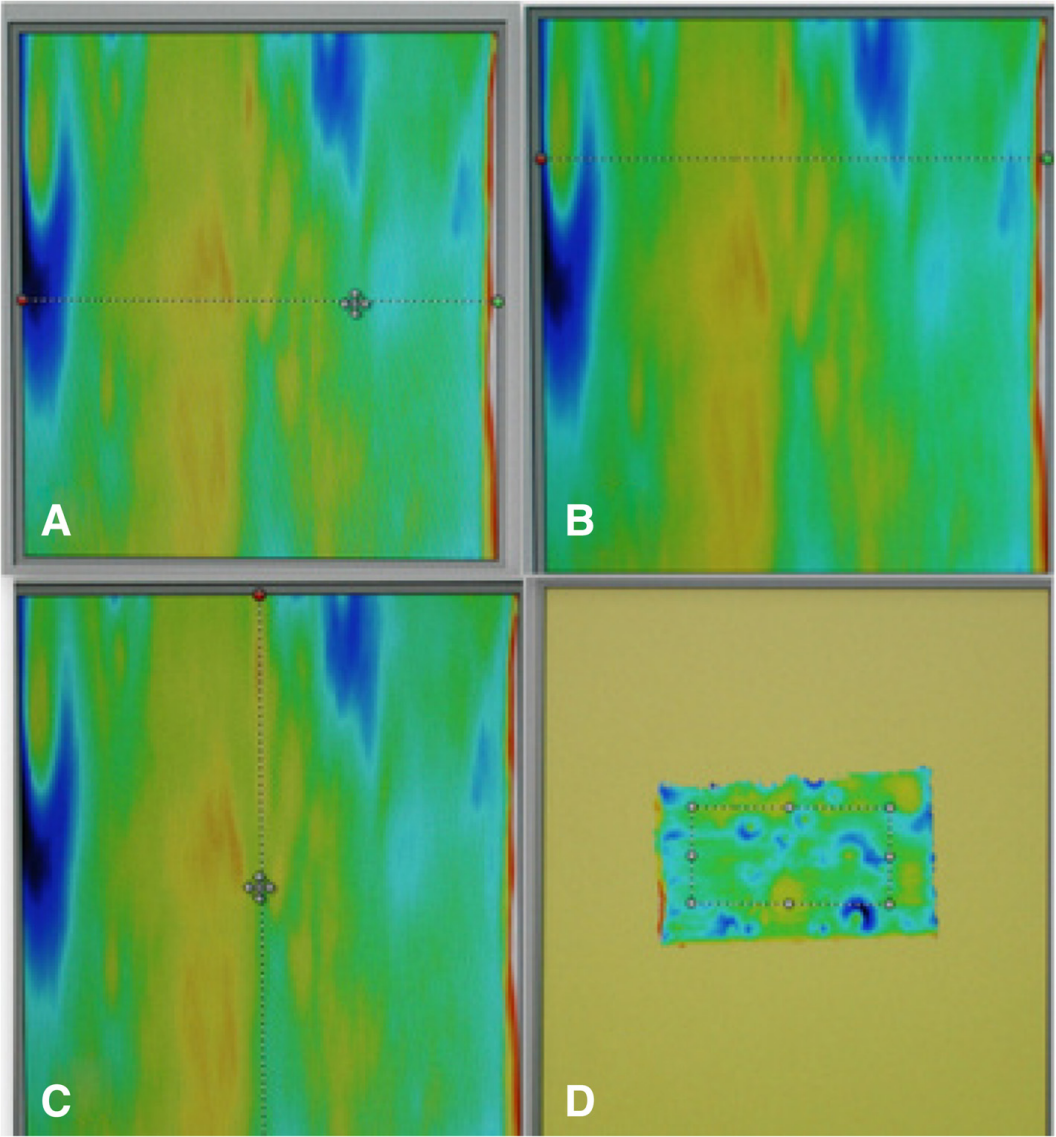
Showing images of the bracket slot. **a**–**c** The profile lines indicate average roughness (Ra) which is two-dimensional. **d** The marked rectangular area as a whole indicates the average surface roughness (Sa) which is three-dimensional

From the point of two-dimensional parameters, Ra value is of significant importance as it is the mean of the roughness profile. But the Ra value represents the slot in two dimensions. If we draw several lines (profile lines) on the area of the bracket slot floor, we will receive several readings for a single slot of the bracket, which means each bracket slot will have many different Ra values, which is not the case in the three-dimensional Sa value, as it is the average of the whole surface area, the area as a whole which is taken into consideration (rectangular dotted line Fig. 7d).

A template is made (Fig. 6) in which once the slot surface is scanned, a raw surface view of the slot is received. The raw surface view which was obtained requires a levelling to be done, so that the whole surface is levelled in one form with the help of the software Digital Surf, (TalyMap Platinum software, Leicester, England, Version no. 6.1.6001). Once this is achieved and the threshold is applied, the software eliminates the highest peak and the deepest valley of the slot floor surface and gives the readings which are less biassed by these highest peak and the deepest valley.

An ICC test between examiners was performed. Twenty percent of the samples from each group were re-evaluated in order to evaluate the error of the method.

Likewise, all the 80 samples of stainless steel bracket measurements were completed. One hundred sixty readings of the Sa value were obtained (Table 3).

**Table 1:** One-way ANOVA test for slot surface roughness

		Sum of squares	*df*	Mean square	*F*	Sig.
Sa_VALUE_MESIAL_SLOT	Between groups	56.26663	3	18.75554	6.898479	0
	Within groups	206.6283	76	2.718794		
	Total	262.895	79			
Sa_VALUE_DISTAL_SLOT	Between groups	90.1959	3	30.0653	16.0508	0
	Within groups	142.3581	76	1.873133		
	Total	232.554	79

**Table 2:** Results of Tukey’s test for slot surface roughness

	(I) group	(J) group	Mean difference (I–J)	Std. error	Sig.	Upper bound	Lower bound
Sa_VALUE_MESIAL_SLOT	Group A 3M Unitek	Group B Ormco^a^	1.66010	0.52142	0.01110	0.29043	3.02977
		Group C Ortho Organizer	0.49775	0.52142	0.77542	−0.87192	1.86742
		Group D American Orthodontics^a^	2.06675	0.52142	0.00093	0.69708	3.43642
	Group B Ormco	Group A 3M Unitek^a^	−1.66010	0.52142	0.01110	−3.02977	−0.29043
		Group C Ortho Organizer	−1.16235	0.52142	0.12463	−2.53202	0.20732
		Group D American Orthodontics	0.40665	0.52142	0.86334	−0.96302	1.77632
	Group C Ortho Organizer	Group A 3M Unitek	−0.49775	0.52142	0.77542	−1.86742	0.87192
		Group B Ormco	1.16235	0.52142	0.12463	−0.20732	2.53202
		Group D American Orthodontics^a^	1.56900	0.52142	0.01827	0.19933	2.93867
	Group D American Orthodontics	Group A 3M Unitek^a^	−2.06675	0.52142	0.00093	−3.43642	−0.69708
		Group B Ormco	−0.40665	0.52142	0.86334	−1.77632	0.96302
		Group C Ortho Organizer^a^	−1.56900	0.52142	0.01827	−2.93867	−0.19933
Sa_VALUE_DISTAL_SLOT	Group A 3M Unitek	Group B Ormco^a^	2.39840	0.43280	0.00000	1.26153	3.53527
		Group C Ortho Organizer^a^	1.39785	0.43280	0.00970	0.26098	2.53472
		Group D American Orthodontics^a^	2.72935	0.43280	0.00000	1.59248	3.86622
	Group B Ormco	Group A 3M Unitek^a^	−2.39840	0.43280	0.00000	−3.53527	−1.26153
		Group C Ortho Organizer	−1.00055	0.43280	0.10433	−2.13742	0.13632
		Group D American Orthodontics	0.33095	0.43280	0.87006	−0.80592	1.46782
	Group C Ortho Organizer	Group A 3M Unitek^a^	−1.39785	0.43280	0.00970	−2.53472	−0.26098
		Group B Ormco	1.00055	0.43280	0.10433	−0.13632	2.13742
		Group D American Orthodontics^a^	1.33150	0.43280	0.01512	0.19463	2.46837
	Group D American Orthodontics	Group A 3M Unitek^a^	−2.72935	0.43280	0.00000	−3.86622	−1.59248
		Group B Ormco	−0.33095	0.43280	0.87006	−1.46782	0.80592
		Group C Ortho Organizer^a^	−1.33150	0.43280	0.01512	−2.46837	−0.19463

^a^The mean difference is significant at the 05 level

**Table 3:** Average surface roughness (Sa values) of bracket slot (measurement in micrometre)

Group A—3M Unitek		Group B—Ormco		Group C—Ortho Organizer		Group D—American Orthodontics	
Mesial slot (A)	Distal slot (B)	Mesial slot (A)	Distal slot (B)	Mesial slot (A)	Distal slot (B)	Mesial slot (A)	Distal slot (B)
1.111	0.968	0.371	1.127	1.85	2.459	0.807	0.852
7.875	6.011	2.162	0.548	3.572	2.107	0.663	0.917
0.52	0.778	1.111	1.291	1.834	3.12	0.034	0.298
7.522	7.899	0.446	0.341	2.148	2.132	0.769	0.717
10.329	5.788	1.427	1.472	2.095	1.934	0.363	0.641
0.814	0.813	0.456	0.701	2.32	1.96	0.877	1.09
0.487	1.122	0.652	0.502	2.401	2.346	0.433	0.292
2.055	5.619	2.454	2.242	2.349	1.516	0.722	0.687
0.652	1.117	0.426	0.369	2.205	2.332	1.704	1.537
3.378	3.751	0.786	0.771	2.781	1.951	1.07	1.124
0.921	1.636	0.423	0.683	2.386	1.805	0.423	0.446
0.531	3.801	0.528	0.292	2.198	1.993	0.425	0.387
5.532	3.773	0.601	0.597	3.215	2.171	0.805	0.999
1.1	6.528	2.46	1.743	1.819	2.228	1.109	1.121
0.741	0.661	0.53	0.35	1.799	2.058	0.34	0.403
0.457	1.425	2.736	3.228	1.797	2.163	0.656	0.917
4.971	6.754	0.531	0.42	2.863	1.622	1.181	0.624
6.007	4.548	0.803	0.521	2.184	1.401	1.006	0.833
0.695	0.789	0.426	0.335	1.886	1.521	0.526	0.522
0.393	5.74	3.56	4.02	2.434	2.745	0.843	0.527

### Method of statistical analysis

1. SEM analysis—images were analysed, using a discrete scale quantitative classification containing four scores.
2. Surface roughness analysis—all the data collected had normal distribution, so we could apply the parametric tests. Analysis of variance (ANOVA) was used to determine whether the difference between the groups was significant or not.
3. Post hoc comparison test (Tukey’s multiple comparison test) was used for inter-group comparison.

## Results

### Scanning electron microscope results

Surface roughness scores were group A = 0—very rough surface, group C = 1—rough surface, group B = 2—smooth surface, and group D = 3—very smooth surface, respectively. This suggests that group D bracket slot floor were smoother as compared to group A, group B, and group C, respectively.

### Surface roughness results

**Fig. 8:**
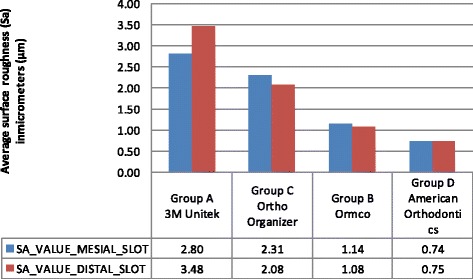
Average surface roughness (Sa) mean values in micrometre across the four groups

Figure 8 shows the mean of the Sa values across the four groups, the surface roughness for both mesial and distal slots was highest in group A, followed by C, B and D, respectively. This indicates that there were differences in the surface roughness across the four groups with group D having the smoothest surface with values 0.74 and 0.75 for the mesial and distal slots, respectively.

Table 1 shows the results of the ANOVA test. From the results, we noticed that the F-statistic of the average surface roughness for the mesial slot was 6.898479 and for the distal it was 16.0508, both of these values result in a very low *p* value (<0.001) which allows us to reject the null hypothesis and conclude that a difference amongst the means across the four groups was present.

Table 2 shows the results of the Tukey’s multiple comparison test (post hoc test). Thus, surface roughness was highest for group A, followed by C, B and D, respectively. Groups B and D provided smooth surface roughness; however, group D had the very smooth surface with values 0.74 and 0.75 for the mesial and distal slots, respectively.

## Discussion

Resistance to sliding (RS) between brackets and archwires during treatment greatly influences the force transmitted to the teeth; to close the extraction space, sliding mechanics are widely used, and they may reduce the orthodontic force as much as 50 % of the bracket [9] and archwire materials. The coefficient of friction is an important factor in RS [10, 11], which might depend on the roughness, texture, and/or hardness of the contacting material surfaces [12]. Thus, studies of bracket surface roughness are of great clinical interest.

Previous studies have measured the surface roughness of brackets and archwires using scanning electron microscopy [13, 14], a contact surface profilometer [15, 16] and atomic force microscopy [11, 15, 17–19] (AFM). SEM can visualize two dimensionally the surface morphology, and a quantitative information is not being provided regarding the selected area. A contact profilometer allows two-dimensional determination of surface roughness parameter values. However, the sample surface adjacent to the scanning line may be damaged as the measured area is in the form of a line, and in contrast, more advantages are being provided by AFM, such as 3D quantitative and configuration measurements of the selected surface. A 3D non-contact surface profilometer which is available and is based on white light interferometry methods uses He-Ne beam of 633 nm [8] which can successfully allow determination and 3D imaging of surface roughness parameter values. The measurement needs no sample preparation and is non-destructive. So, the test range cover of ∼10 mm can be achieved. So, the surface roughness of the all the specimens were evaluated by using SEM and 3D interferometry profilometer machines, which is non-destructive and much faster compared with a stylus profilometer, and with a larger field, needing no sample preparation, compared with AFM [6].

Hence, all the tests were carried out to analyse quantitatively the morphological surface of the bracket slot floor with the help of SEM machine and to qualitatively analyse the Sa of the bracket slot floor with the help of the 3D non-contact optical surface profilometer machine. In the present study, it was found that the F-statistic of average surface roughness for the mesial slot was 6.898479 and for the distal, it was 16.0508 both of these values result in a very low *p* value (<0.001) which concluded that a difference amongst the means across the four groups was present. The surface roughness was highest for group A, followed by C, B and D, respectively. Groups B and D provided smooth surface roughness; however, group D had the very smooth surface with values 0.74 and 0.75 for the mesial and distal slots, respectively.

Ceramic brackets, whether they are single crystal sapphire, polycrystalline alumina, or zirconia, relative to stainless steel (SS) brackets, have poor frictional properties [13, 20, 21]. To combat this, aesthetic ceramic brackets with metallic slot have been developed [22, 23]. Compared to stainless steel brackets, titanium brackets have more surface roughness [24, 25]. Self-ligating brackets [26–28] with low-friction and full bracket engagement of the archwire, which is easy to use, assist good oral hygiene and is comfortable for the patient. But because of its partially reduced torquing capacity and high cost, conventional brackets are widely being used [29]. The above studies reveal that conventional metallic brackets are widely used than ceramic, zirconia, titanium, and self-ligating brackets either due to their properties or cost factor. Hence, the present study evaluated the conventional stainless steel brackets.

The present study was conducted to analyse the Sa of the bracket slot floor of the conventional stainless steel bracket with the help of the 3D non-contact optical surface profilometer machine. The surface roughness was highest for group A, followed by C, B and D, respectively. Groups B and D provided smooth surface roughness; however, group D had the very smooth surface with values 0.74 and 0.75 for the mesial and distal slots, respectively.

In order to select the proper low-friction bracket system, clinicians should consider specific characteristics of slot design [30]. MBT recommends 0.022-in.-slot height than 0.018-in.-slot height [31]. Regarding the use of 0.022-in.-slot, 54 % of orthodontists preferred the 0.022-in.-slot size [32]. With this view, we decided to take 0.022-in. MBT slot conventional stainless steel brackets for the present study. Upper-right first premolar brackets were included in this study as to maintain a standardized protocol.

Many previous studies [1, 2, 6, 17, 19, 20, 24, 31–37] have been done on surface roughness. Some of these studies evaluated the friction characteristics, surface roughness, ligation method, etc. To evaluate friction, there is a need to compare any two materials like stainless steel, ceramic, either mono or polycrystalline, titanium, and zirconium. But the present study evaluated the surface roughness of stainless steel brackets itself with the help of the 3D non-contact optical surface profilometer machine and SEM. There was no comparison of friction of the stainless steel brackets with the other materials like wires or ligation technique. So the study was focussed on evaluating the surface roughness in three dimensions of the stainless steel bracket slot which can help to determine the clinical performance of the bracket, the accuracy of bracket slot dimension and bracket slot roughness. The brackets Mini 2000, Ormco Corp. (Glendora, CA) and Mini master (American Orthodontics) have smooth surfaces of bracket slot.

The effects of the oral environment cannot be simulated in an in vitro exploration, and a possible limitation of the present study is the small sample size in each group. In vivo studies with a large sample size in each group will be needed to examine the intraoral exposure effects on surface roughness in three dimensions of the stainless steel bracket slot.

## Conclusions

- On SEM evaluation for surface roughness of bracket slot floor, surface roughness for group D was found to be the smoothest of all the groups, while group B is less smooth, group C is rough, and group A was found to have a very rough surface.
- Measurement of surface roughness of the bracket slot floor with the 3D non-contact surface profilometer machine, led to the conclusion that the average surface roughness was highest for group A, followed by group C, group B and group D, respectively.

## References

[1] Liu X, Lin J, Ding P (2013). Changes in the surface roughness and friction coefficient of orthodontic bracket slots before and after treatment. Scanning.

[2] Doshi UH, Bhad Patil WA (2011). Static frictional force and surface roughness of various bracket and wire combinations. Am J Orthod Dentofacial Orthop.

[3] Proffit WR, Fields HW, Sarver DM (2009). Contemporary orthodontics, 4th ed.

[4] Nanda R (1997). Biomechanics in clinical orthodontics.

[5] Juvvadi SR, Kailasam V, Padmanabhan S, Chitharanjan AB (2010). Physical, mechanical, and flexural properties of 3 orthodontic wires: an in-vitro study. Am J Orthod Dentofacial Orthop.

[6] Dolci GS, Spohr AM, Zimmer ER, Marchioro EM (2013). Assessment of the dimensions and surface characteristics of orthodontic wires and bracket slots. Dental Press J Orthod.

[7] Chappard D, Degasne I, Hure G, Legand E, Audan M, Basle MF (2003). Image analysis of roughness by texture and fractal analysis correlate with contact profilometry. Biomater.

[8] Synak R, Lipinski W, Pawelczak M (2012). A novel method for areal surface roughness measurement. World Acad Sci Eng Tech.

[9] Drescher D, Bourauel C, Schumacher H (1989). Frictional forces between bracket and archwire. Am J Orthod Dentofacial Orthop.

[10] De Franco DJ, Spiller RE, von Fraunhofer JA (1995). Frictional resistance using Teflon-coated ligatures with various bracket-archwire combinations. Angle Orthod.

[11] Choi S, Park KH, Cheong Y, Kim HK, Park YG, Park HK (2011). Changes in ultrastructure and properties of bracket slots after orthodontic treatment with bicuspid extraction. Scanning.

[12] Loftus BP, Artun J, Nicholls JI, Alonzo TA, Stoner JA (1999). Evaluation of friction during sliding tooth movement in various bracket-arch wire combinations. Am J Orthod Dentofacial Orthop.

[13] Saunders CR, Kusy RP (1994). Surface topography and frictional characteristics of ceramic brackets. Am J Orthod Dentofacial Orthop.

[14] Marques IS, Araujo AM, Gurgel JA, Normando D (2010). Debris, roughness and friction of stainless steel archwires following clinical use. Angle Orthod.

[15] Bourauel C, Fries T, Drescher D, Plietsch R (1998). Surface roughness of orthodontic wires via atomic force microscopy, laser specular reflectance, and profilometry. Eur J Orthod.

[16] Zinelis S, Eliades T, Eliades G, Makou M, Silikas N (2005). Comparative assessment of the roughness, hardness, and wear resistance of aesthetic bracket materials. Dent Mater.

[17] Lin MC, Lin SC, Lee TH, Huang HH (2006). Surface analysis and corrosion resistance of different stainless steel orthodontic brackets in artificial saliva. Angle Orthod.

[18] Alcock JP, Barbour ME, Sandy JR, Ireland AJ (2009). Nano-indentation of orthodontic archwires: the effect of decontamination and clinical use on hardness, elastic modulus and surface roughness. Dent Mater.

[19] Lee GJ, Park KH, Park YG, Park HK (2010). A quantitative AFM analysis of nano-scale surface roughness in various orthodontic brackets. Micron.

[20] Pratten DH, Popli K, Germane N, Gunsolley JC (1990). Frictional resistance of ceramic and stainless steel orthodontic brackets. Am J Orthod Dentofacial Orthop.

[21] Keith O, Kusy RP, Whitley JQ (1994). Zirconia brackets: an evaluation of morphology and coefficients of friction. Am J Orthod Dentofacial Orthop.

[22] Angolkar PV, Kapila S, Duncanson MG, Nanda RS (1990). Evaluation of friction between ceramic brackets and orthodontic wires of four alloys. Am J Orthod Dentofacial Orthop.

[23] Ghafari J (1992). Problems associated with ceramic brackets suggest limiting use on selected teeth. Angle Orthod.

[24] Kusy RP, Whitely JQ, Ambrose WW, Newman JG (1998). Evaluation of titanium brackets for orthodontic treatment: part I. The passive configuration. Am J Orthod Dentofacial Orthop.

[25] Kusy RP, O’Grady PW (2000). Evaluation of titanium brackets for orthodontic treatment: part II. The active configuration. Am J Orthod Dentofacial Orthop.

[26] Kusy RP, Whitely JQ (1989). Effects of sliding velocity on coefficients of friction in a model orthodontic system. Dent Mater.

[27] Birnie D, Harradine N (2008). Self-ligating orthodontic brackets. Semin Orthod.

[28] Hain M, Dhopatkar A, Rock P (2006). A comparison of different ligation methods on friction. Am J Orthod Dentofacial Orthop.

[29] Cacciafesta V, Sfondrini MF, Ricciardi A, Scribante A, Klersy C, Auricchio F (2003). Evaluation of friction of stainless steel and esthetic self-ligating brackets in various bracket-archwire combinations. Am J Orthod Dentofacial Orthop.

[30] Nucera R, Giudice LA, Matarese G, Artemisia A, Bramanti E, Crupi P, Cordasco G (2013). Analysis of the characteristics of slot design affecting resistance to sliding during active archwire configurations. Prog in Orthod.

[31] McLaughlin RP, Bennett JC, Trevisi HJ (2002). Systemized orthodontic treatment mechanics.

[32] Lombardo L, Wierusz W, Toscano D, Lapenta R, Kaplan A, Siciliani G (2013). Frictional resistance exerted by different lingual and labial brackets: an in vitro study. Prog in Orthod.

[33] Keim RG, Gottlieb EL, Nelson AH, Vogel DS (2002). 2002 JCO study of orthodontic diagnosis and treatment procedures: part 1: results and trends. J Clin Orthod.

[34] Subbiah S, Balasubramanian RK, Raj PK, Dilip S (2010). Comparison of frictional resistance between conventional stainless steel, metal insert ceramic, self-ligating stainless steel and self ligating ceramic brackets with stainless steel wire: in vitro study. J Ind Orthod Soc.

[35] Faizee KMSH, Thomas S, Krishnaswamy NR (2011). Frictional characteristics of active and passive self-ligation bracket systems: an in vitro study. J Ind Orthod Soc.

[36] Galvão BM, Camporesi M, Tortamano A, Dominguez GC, Defraia E (2013). Frictional resistance in monocrystalline ceramic brackets with conventional and nonconventional elastomeric ligatures. Prog in Orthod.

[37] Choi SH, Kang DY, Hwang CJ (2014). Surface roughness of three types of modern plastic bracket slot floors and frictional resistance. Angle Orthod..

